# Anorectal Transplantation in Human Cadavers: Mock Anorectal Allotransplantation

**DOI:** 10.1371/journal.pone.0068977

**Published:** 2013-07-11

**Authors:** Jun Araki, Yuji Nishizawa, Tomoyuki Sato, Munekazu Naito, Keiichi Akita, Kensuke Tashiro, Takuya Iida, Isao Koshima

**Affiliations:** 1 Department of Plastic Surgery, University of Tokyo, Tokyo, Japan; 2 Department of Gastroenterological Surgery, Faculty of Medicine, Kagawa University, Kagawa, Japan; 3 Saitama Shinkaibashi Clinic, Saitama, Japan; 4 Department of Anatomy, Tokyo Medical University, Tokyo, Japan; 5 Department of Clinical Anatomy, Tokyo Medical and Dental University, Tokyo, Japan; University of Colorado School of Medicine, United States of America

## Abstract

**Background:**

Anorectal transplantation is a method for patients who have lost their anorectal function or suffer from congenital anorectal dysfunction to recover this function, and this has been investigated in experimental animal models using pigs, dogs, and rats. In this study, we performed an examination of anorectal transplantation in human cadavers to investigate whether this procedure could be performed in patients.

**Methods:**

A 77-year-old woman cadaver 1 was used as the donor and a 98-year-old woman cadaver 2 was used as the recipient. Initially, abdominoperineal excision of the anus and rectum (the Miles’ operation) was performed on the recipient. Next, an anorectal graft containing the pudendal nerve (PN), pudendal artery (PA), pudendal vein (PV), inferior mesenteric artery (IMA), and inferior mesenteric vein (IMV) was harvested from the donor. The donor graft was transplanted into the recipient by intestinal anastomosis and microneurovascular anastomoses orthotopically.

**Results:**

The diameters of the PN (right/left), IMA, and IMV were 2.5 mm/2.5 mm, 2.0 mm, and 1.5 mm, respectively, in cadaver 1, and 2.0 mm/2.0 mm, 2.0 mm, and 2.0 mm, respectively, in cadaver 2. The length of the PN, PA, PV, IMA, and IMV in the graft was sufficient to allow proper anastomosis.

**Conclusion:**

This preliminary study indicated that human anorectal transplantation was possible anatomically and technically. We anticipate our study will aid in the potential future application of this procedure to human patients.

## Introduction

Anal function is often lost due to resections of rectal or perianal cancer, congenital anal dysfunctions, such as anal atresia or Hirschsprung’s disease, intractable anal fistulas of inflammatory bowel diseases, such as seen in Crohn’s disease or ulcerative colitis, and other severe incontinences, such as seen after accidental trauma or perineal laceration from childbirth. In addition, current treatments for severe fecal incontinence are often complex and have unsatisfactory results [1]. Colostomies have served as effective surgery for various anorectal dysfunctions and play an important role in allowing excretion in patients. However, it must be noted that these patients suffer greatly from stresses caused by their stoma. Patients have reported decreased sexual activity and fertility at the time of survey due to colectomy, especially for females [2], and occasionally prepared for death rather than accepted their condition and potential cure [3]. To avoid colostomies, various anal reconstructions have been performed and many alternative therapies have been examined and developed [4–6]; however, no alternative options have been able to solve this critical issue until now.

Anorectal transplantation is a supreme method for patients who have lost their anorectal function or suffer from congenital anorectal dysfunction to recover this function, and this has been investigated in experimental animal models [7–12]. Experimental anorectal transplantation was first attempted at St. Mark’s Hospital, UK, in 2000 [7]. In that study, the anorectum from female pigs was transplanted into male pigs without immunosuppression and successful inferior mesenteric artery (IMA), inferior mesenteric vein (IMV), and pudendal nerve (PN) anastomoses were achieved. Since then, similar experimental studies have been reported using rat [8–10], swine [11], and canine models [12]. However, no reports currently exist in the literature examining the possibility of anorectal transplantation in humans. Recently, laparoscopic surgery of the rectum (e.g., intersphincteric resection) has allowed the surgical anatomy of the pelvis to be clearer than open surgery [13,14], and pelvic anatomy in cadaver studies has improved because of advances in techniques for anorectal surgery [15,16]. These anatomical understandings may cause secure reconstruction of the pelvic floor. The aim of this study was to examine whether anorectal transplantation was possible in patients by performing mock anorectal transplantation in human cadavers.

## Methods

### Information on Cadavers

The cadavers used in this study were donated to the Department of Anatomy, Tokyo Medical and Dental University, Tokyo, Japan. Before they died, they made documents of agreement of donation of the body and those of agreement of use for the clinical studies. The format of the document is within expectation of the Japanese law “Act on Body Donation for Medical and Dental Education”.

A 77-year-old woman who died from right renal cancer was used as a donor. A 98-year-old woman who died of natural causes was used as the recipient. Neither of the women had any past surgical history in their buttocks, colons, rectums, and anuses. All cadavers had been embalmed using the method described by Thiel [17,18]. Thiel cadavers are embalmed in a water-based solution of salts for fixation, boric acid for disinfection, glycol, chlorocresol and ethanol, and a very small amount of formaldehyde. This precipitation leads to homogenization of the tissue. The physiologic texture of the tissue is maintained by further effects of precipitation and linking caused by the embalming solution. The skin is life-like, joints are fully flexible, and ultrasound imaging of nerves and needle guidance is realistic.

### Mock Anorectal Transplantation in Human Cadavers

Two colorectal surgeons, one plastic surgeon, and one anatomist performed the mock anorectal transplantation together. Initially, abdominoperineal excision (Miles’ operation) was performed on the recipient. Next, an anorectal graft was harvested from the donor, and then the donor graft was transplanted into the recipient.

## Results

### Technique of Recipient Abdominoperineal Excision (Miles’ operation)

The other cadaver served as the recipient of the anorectal transplants (mock transplants). To mimic the clinical situation, an abdominoperineal excision (the Miles’ operation) was performed. Briefly, the procedure was performed through two incisions, one in the abdomen and one through the region between the anus and the genitals. Surgery proceeded from the anterior abdominal incision to the perineal incision. In the abdomen, the surgeon followed the plane outside the mesorectum down to the pelvic floor to the top of the anal canal, and the mesorectum was mobilized from the levator muscles. The perineal part of the operation was then performed from below in the prone position, with excision of the anal canal including the surrounding skin, ischiorectal fat, and the upper portions of the levator muscles. The anal canal was circumferentially dissected outside of the external anal sphincter muscle. Pudendal nerves (PNs), pudendal arteries (PAs), and pudendal veins (PVs) ran bilaterally along the inside of the ischial tuberosity and reached the external anal sphincter muscle. The posterior wall of the anal segment was separated from the anterior surface of the coccygeal muscle. The levator ani muscle was transected at the lateral and posterior wall, and the anterior wall was detached behind the vagina. The rectum was separated with mesentery at the lower part. The IMA, IMV, PAs, PVs, and PNs were clipped and cut. Finally, the anal segment was resected.

### Technique of Donor Anorectal Graft Harvesting

**Figure 1:**
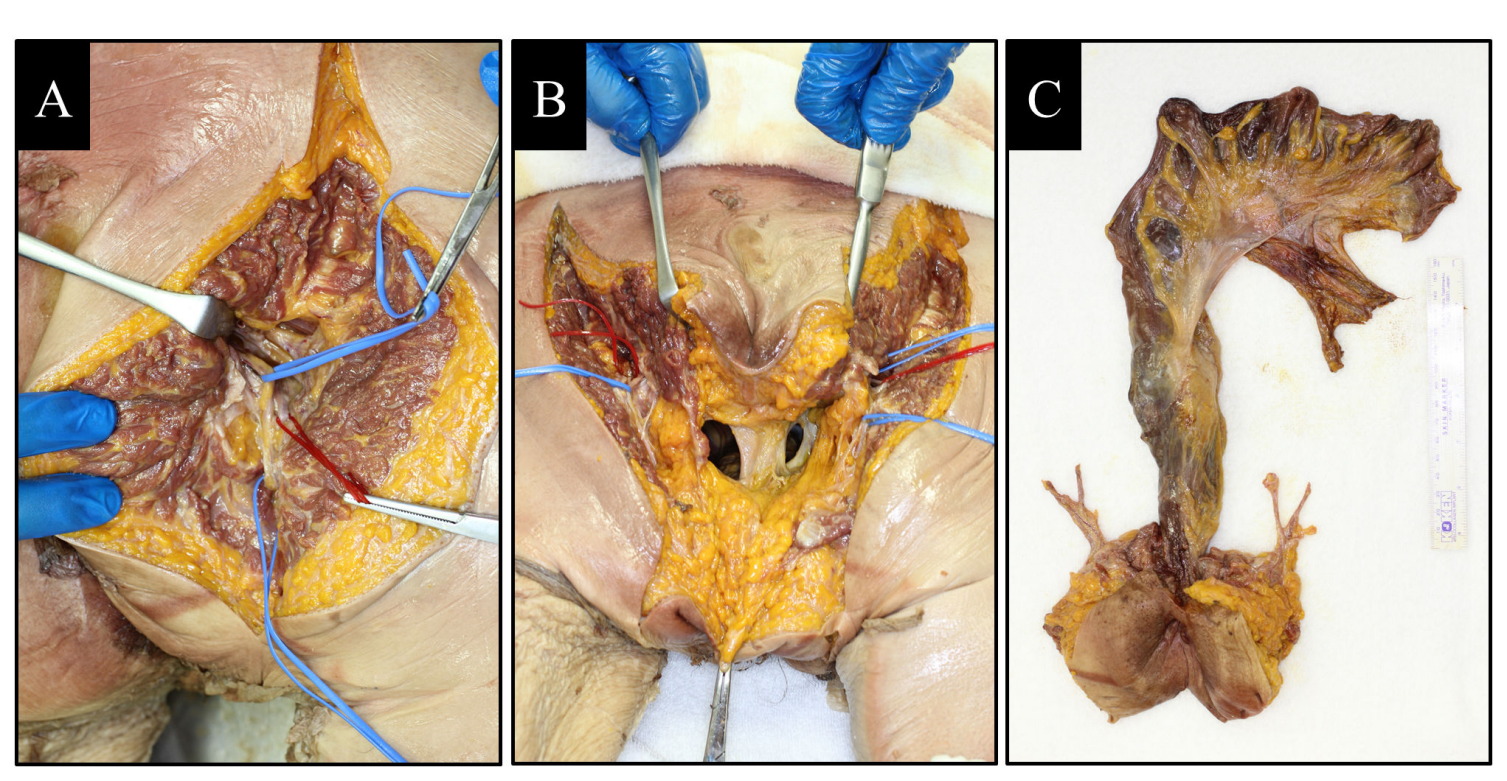
Donor anorectal graft harvesting. (A) Right pudendal nerves (PN) and pudendal artery (PA) and vein (PV) were separated by the blue (PN) and red (PA and PV) vessel loop. (B) Pelvic floor muscles were identified and cut. (C) The anorectal graft was harvested.

One cadaver served as the donor of the anorectal transplantation (deceased donor). Surgery proceeded from the anterior abdominal incision to the perineal incision. In the abdomen, the surgeon followed the plane outside the mesorectum down to the pelvic floor to the top of the anal canal, and the mesorectum was mobilized from the levator muscles. Next, the gluteal incision was performed in a prone position. The neurovascular bundle, including the PN and pudendal artery (PA) and vein (PV), ran through the pudendal canal, and this was divided and marked (Figure 1-A). The dissection of the deeper layer reached into the pelvis and pelvic floor muscles were identified (Figure 1-B) and cut. Next, the perineal part of the operation was performed from below in the prone position, with excision of the anal canal including the surrounding skin, ischiorectal fat, and the upper portions of the levator muscles. The anal canal was circumferentially dissected outside of the external anal sphincter muscle into the pelvis. After clipping and cutting of the IMA, IMV, PNs, PAs, and PVs, the anorectal graft was finally harvested (Figure 1-C).

### Technique of Donor Anorectal Graft Transplantation into the Recipient’s Anorectal Defect

**Figure 2:**
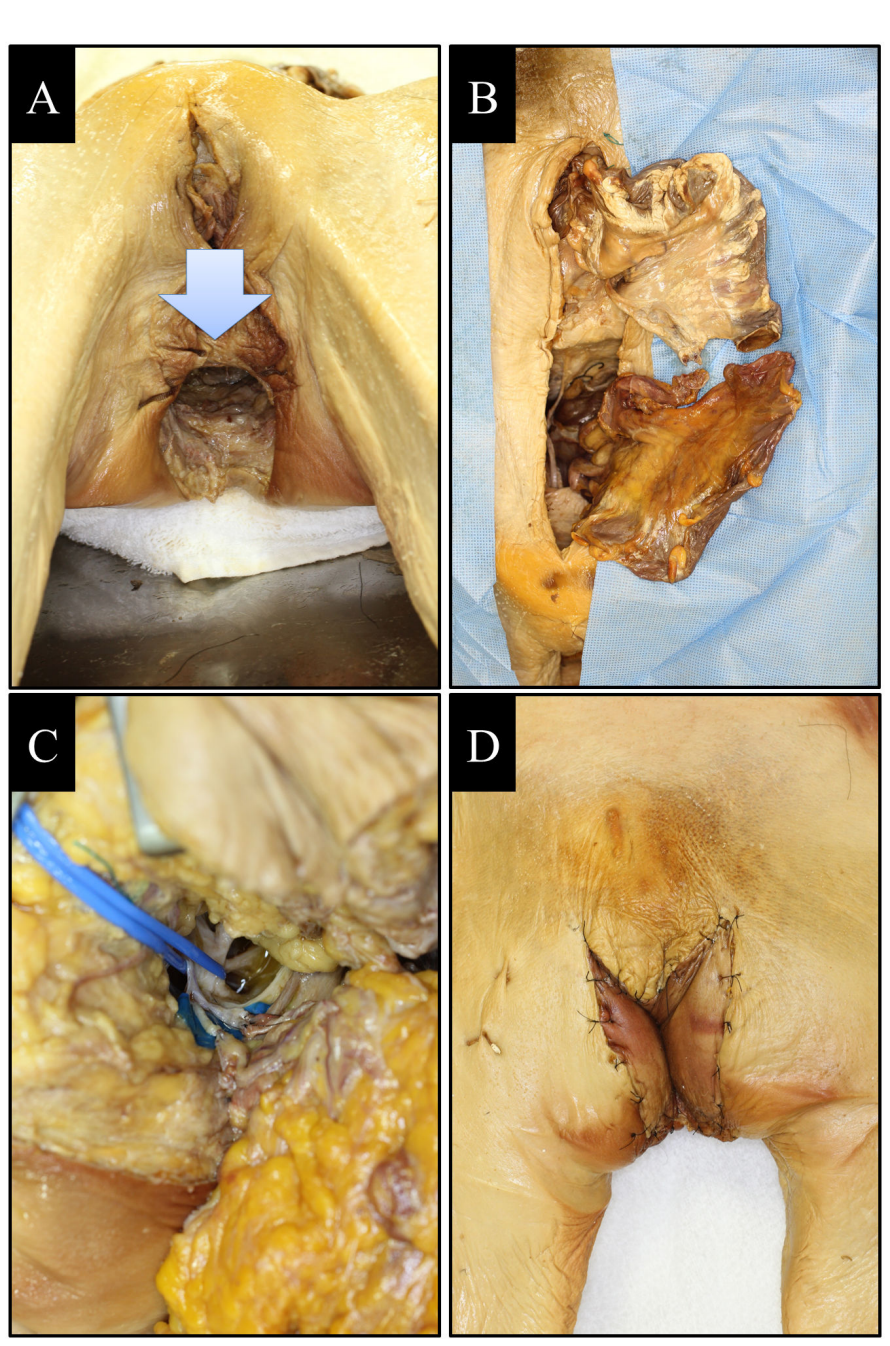
Donor anorectal graft transplantation into the recipient’s anorectal defect. (A) Recipient anorectal defect (blue arrow) after abdominoperineal excision (Miles’ operation). (B) The IMA, IMV, and intestine of the donor graft were anastomosed to those of the recipient outside of the abdominal cavity. (C) The branches of the pudendal nerve (PN) were anastomosed to the graft’s PNs with epineural sutures. (D) The skin incisions were closed and the inset of the flap was completed.

After anorectal graft harvest, the created defect in the recipient consisted of the anorectal defect (Figure 2-A). The harvested anorectal graft was transferred from the donor to the recipient and the sequence of the graft inset was as follows. The inset of the donor graft started with reconstruction of the pelvic floor region in the supine position. The pelvic floor muscles and ligaments were strongly sutured to prevent descent of the graft. Next, the proximal end of the recipient intestine was anastomosed to the distal end of the donor intestine using the Albert-Lambert method. Next, the branches of the IMV and IMA were anastomosed end-to-end with interrupted sutures (8-0 nylon, Johnson & Johnson, Tokyo, Japan) outside of the abdominal cavity (Figure 2-B). Then, the anorectal graft was orthotopically repositioned and the abdomen was closed. Next, in the prone position, the recipient’s circumanal incision was extended to the buttock, and the PNs were bilaterally identified under the gluteus maximus muscle. The neural branches suspected to be proceeding to the anal sphincter muscle were anastomosed to the graft’s PNs with epineural sutures (Figure 2-C). Finally, the skin incisions were closed and the inset of the flap was completed (Figure 2-D).

### Operative Duration and Diameters of Nerves and Vessels

The duration of the surgical procedure was approximately seven hours: three hours for donor anorectal graft harvesting, two hours for the recipient abdominoperineal excision, and two hours for transplantation into the defect. The diameters of the PN (right/left), IMA, and IMV were 2.5 mm/2.5 mm, 2.0 mm, and 1.5 mm, respectively, in cadaver 1, and 2.0 mm/2.0 mm, 2.0 mm, and 2.0 mm, respectively, in cadaver 2. A summary of the data from the cadavers is presented in Table 1.

**Table 1:** Case summary and surgical parameters in human cadavers.

	Cadaver 1	Cadaver 2
Age at death	77	98
Sex	Female	Female
Model	Donor	Recipient
Cause of death	Right renal cancer	Natural cause
Time for donor arorectal graft harvesting	3 h	-
Time for recipient abdominoperineal excision	-	2 h
Diameter of pudendal nerve (right/left)	2.5 mm/2.5 mm	2.0 mm/2.0 mm
Diameter of inferior mesenteric artery	2.0 mm	2.0 mm
Diameter of inferior mesenteric vein	1.5 mm	2.0 mm

## Discussion

Anorectal function is constituted in a composite manner by various structures such as the rectum, anal canal, levator ani, and anal sphincter muscle, which is innervated by PNs. The muscular transfer of the gracilis [4] or the gluteus maximus muscles [5] and the implantation of an artificial sphincter [6] have previously been performed to recover anal function. However, none of these procedures are considered ‘gold standard’ techniques due to the complex nature of anorectal functions. Anorectal transplantation seems to be a supreme method allowing for reconstruction of anal function, which is advantageous over other methods. This is the first report describing mock anorectal transplantation in a human cadaver. Since two human cadavers were fixed by the Thiel method, the flexibility of the joints was well preserved and it was easy to take the appropriate positions for the operation.

In this study, the IMA, IMV, PNs, PAs, and PVs were obviously detected in both the donor and recipient cadavers (Figure 1-A, B Figure 2-C). The length and diameter of the IMA, IMV, PNs, PAs, and PVs were sufficient to allow anastomoses without tension. Our previous experiment in dogs revealed that the anastomoses of PNs, PAs, and PVs had an important role in the success of anorectal transplantation [12]. A previous study in humans described the length of the PNs passing out below the sacrotuberous ligament as being varied from 21 to 44 mm (median, 29.5 mm) [19]. The diameter of the PAs in our human cadaver was thinner than the previously reported human data (2.7 ± 0.4 mm at right and 2.7 ± 0.5 mm at left) [20]. This could be because in cadavers, vessels are collapsed and lack elasticity and turgor, and nerves are easily dried during dissection [21]. These data indicated that the anastomoses of the IMA, IMV, PNs, PAs, and PVs were technically possible in human anorectal transplantation. In addition, the levator ani muscle could be strongly sutured to the pelvic wall to avoid graft ptosis. In this initial study, the warm ischemia time was about one hour from vessel clamps of the IMA and IMV to de-clamp of them after anastomoses. We anticipate that it will also be about one hour in clinical practice and the cold ischemia time including perfusion and transport will take in total ischemia time. The warm/cold ischemia time in intestinal transplantation is about 40 minutes/ 7 hours [22]. The ischemia times of other composite tissue allografts were 5 h 10 min (cold) in hand [23], 2 h and 40 min in face [24], and 10 hours (cold) in larynx [25]. These times will fit within the range of acceptable ischemia time for anorectal transplantation. As a result, we performed mock anorectal transplantation from a deceased donor into a recipient after abdominoperineal excision.

The clinical application of anorectal transplantation is not only limited to anal dysfunction resultant from post-abdominoperineal excision, but also that caused by anal atresia, Hirschsprung’s disease, intractable anal fistulas, or trauma [26]. And still farther, it may be accompanied en bloc colon and small bowel transplantation [27]. Therefore, it is necessary to develop appropriate transplantation and reconstruction methods for various defects. Evidently, the postoperative anorectal function must also be examined in detail. It is well known that the PVs provide the majority sensations and functions of the anorectum [28]. In our preliminary study using dogs, anorectal function recovery could be observed at six months after re-anastomoses of the PVs (data not shown). And also, the use of immunosuppressants is required to control transplant infection and rejection. To confirm these, it is necessary to perform long-term observations after anorectal allotransplantation in experimental animals such as dogs or monkeys. It has been thought that anorectal transplantation is practically difficult in the clinical setting. However, considering recent advances in operational techniques and transplantation medicine, anorectal transplantation may follow the success of other vascularized composite allotransplants in humans, including those of the limb [24], face [25], larynx [26], and uterus [29]. The ethics of non-life-saving organ transplants involves an assessment of a possible gain in the quality of life for the recipient relative to the risks of the procedure and the follow-up interventions necessary for maintaining organ function [30].

## References

[1] Wald A (2007) Clinical practice. Fecal incontinence in adults. N Engl J Med 356: 1648-1655. doi:10.1056/NEJMcp067041. PubMed: 17442907. 17442907 10.1056/NEJMcp067041

[2] Dalal DH, Patton D, Wojcicki JM, Clark AL, Garnett EA et al. (2012) Quality of life in patients postcolectomy for pediatric-onset ulcerative colitis. J Pediatr Gastroenterol Nutr 55: 425-428. doi:10.1097/MPG.0b013e318253f2f0. PubMed: 22437468. 22437468 10.1097/MPG.0b013e318253f2f0 PMC4446343

[3] Hurny C, Holland J (1985) Psychosocial sequelae of ostomies in cancer patients. CA Cancer J Clin 35: 170-183. doi:10.3322/canjclin.35.3.170. PubMed: 3921203. 3921203 10.3322/canjclin.35.3.170

[4] Chapman AE, Geerdes B, Hewett P, Young J, Eyers T et al. (2002) Systematic review of dynamic graciloplasty in the treatment of faecal incontinence. Br J Surg 89: 138-153. doi:10.1046/j.1365-2168.2002.02018.x. PubMed: 11856125. 11856125 10.1046/j.0007-1323.2001.02018.x

[5] Sato T, Konishi F, Endoh N, Uda H, Sugawara Y et al. (2005) Long-term outcomes of a neo-anus with a pudendal nerve anastomosis contemporaneously reconstructed with an abdominoperineal excision of the rectum. Surgery 137: 8-15. doi:10.1016/S0039-6060(05)00092-9. PubMed: 15614275. 15614275 10.1016/j.surg.2004.05.046

[6] Vaizey CJ, Kamm MA, Gold DM, Bartram CI, Halligan S et al. (1998) Clinical, physiological, and radiological study of a new purpose-designed artificial bowel sphincter. Lancet 352: 105-109. doi:10.1016/S0140-6736(97)11427-1. PubMed: 9672276. 9672276 10.1016/s0140-6736(98)85014-9

[7] O’Bichere A, Shurey S, Sibbons P, Green C, Phillips RK (2000) Experimental model of anorectal transplantation. Br J Surg 87: 1534-1539. doi:10.1046/j.1365-2168.2000.01557.x. PubMed: 11091242. 11091242 10.1046/j.1365-2168.2000.01557.x

[8] Galvão FH, Seid VE, Nunes dos Santos RM, Kitamura M, de Castro Galvão R et al. (2009) Anorectal transplantation. Tech Coloproctol 13: 55-59. doi:10.1007/s10151-009-0459-5. PubMed: 19288244. 19288244 10.1007/s10151-009-0459-5

[9] Galvão FH, Waisberg DR, De Mello Vianna RM, De Castro Galvão R, Seid VE et al. (2012) Intestinal transplantation including anorectal segment in the rat. Microsurgery 32: 77-79. doi:10.1002/micr.20958. PubMed: 22002856. 22002856 10.1002/micr.20958

[10] Araki J, Mihara M, Narushima M, Iida T, Sato T et al. (2011) Vascularized anal autotransplantation model in rats: preliminary report. Transplant Proc 43: 3552-3556. doi:10.1016/j.transproceed.2011.08.042. PubMed: 22099840. 22099840 10.1016/j.transproceed.2011.08.042

[11] Galvao FH, Seid VE, Waisberg DR, Cruz RJ Jr, Hirano H et al. (2012) An innovative model of autologous anorectal transplantation with pudendal nerve reconstruction. Clin (Sao Paulo); 67: 971-972. doi:10.6061/clinics/2012(08)20. 10.6061/clinics/2012(08)20 PMC3416907 22948469

[12] Araki J, Nishizawa Y, Nakamura T, Sato T, Naito M et al. (2012) The development of a canine anorectal autotransplantation model based on blood supply: a preliminary case report. PLOS ONE 7: e44310. doi:10.1371/journal.pone.0044310. PubMed: 22970198. 22970198 10.1371/journal.pone.0044310 PMC3435401

[13] Park JS, Choi GS, Jun SH, Hasegawa S, Sakai Y (2011) Laparoscopic versus open intersphincteric resection and coloanal anastomosis for low rectal cancer: intermediate-term oncologic outcomes. Ann Surg 254: 941-946. doi:10.1097/SLA.0b013e318236c448. PubMed: 22076066. 22076066 10.1097/SLA.0b013e318236c448

[14] Lim SW, Huh JW, Kim YJ, Kim HR (2011) Laparoscopic intersphincteric resection for low rectal cancer. World J Surg 35: 2811-2817. doi:10.1007/s00268-011-1277-2. PubMed: 21959930. 21959930 10.1007/s00268-011-1277-2

[15] Kinugasa Y, Arakawa T, Abe H, Abe S, Cho BH et al. (2012) Anococcygeal raphe revisited: a histological study using mid-term human fetuses and elderly cadavers. Yonsei Med J 53: 849-855. doi:10.3349/ymj.2012.53.4.849. PubMed: 22665356. 22665356 10.3349/ymj.2012.53.4.849 PMC3381476

[16] Matsubara A, Murakami G, Arakawa T, Yasumoto H, Mutaguchi K, et al. (2003) Topographic anatomy of the male perineal structures with special reference to perineal approaches for radical prostatectomy. Int J Urol 10: 141-148 10.1046/j.1442-2042.2003.00585.x 12622710

[17] Thiel W (2002) Supplement to the conservation of an entire cadaver according to W Thiel. Ann Anat 184: 267-269. doi:10.1016/S0940-9602(02)80121-2. PubMed: 12061344. 12061344 10.1016/s0940-9602(02)80121-2

[18] Thiel W (1992) The preservation of the whole corpse with natural color. Ann Anat 174: 185-195. doi:10.1016/S0940-9602(11)80346-8. PubMed: 1503236. 1503236

[19] Sato T, Konishi F, Kanazawa K (1997) Functional perineal colostomy with pudendal nerve anastomosis following anorectal resection: a cadaver operation study on a new procedure. Surgery 121: 569-574. doi:10.1016/S0039-6060(97)90113-6. PubMed: 9142157. 9142157 10.1016/s0039-6060(97)90113-6

[20] Rogers JH, Karimi H, Kao J, Link D, Javidan J et al. (2010) Internal pudendal artery stenoses and erectile dysfunction: correlation with angiographic coronary artery disease. Catheter Cardiovasc Interv 76: 882-887. doi:10.1002/ccd.22646. PubMed: 20928837. 20928837 10.1002/ccd.22646

[21] Siemionow M, Agaoglu G, Unal S (2006) A cadaver study in preparation for facial allograft transplantation in humans: part II. Mock facial transplantation. Plast Reconstr Surg 117: 876-885. doi:10.1097/01.prs.0000204876.27481.fc. PubMed: 16525279. 16525279 10.1097/01.prs.0000204876.27481.fc

[22] Takahashi H, Kato T, Selvaggi G, Nishida S, Gaynor JJ et al. (2007) Subclinical rejection in the initial postoperative period in small intestinal transplantation: a negative influence on graft survival. Transplantation 84: 689-696. doi:10.1097/01.tp.0000280541.83994.93. PubMed: 17893601. 17893601 10.1097/01.tp.0000280541.83994.93

[23] Jones JW, Gruber SA, Barker JH, Breidenbach WC (2000) Successful hand transplantation. One-year follow-up. Louisville Hand Transpl Team N Engl J Med 343: 468-473. 10.1056/NEJM200008173430704 10950668

[24] Siemionow M, Papay F, Alam D, Bernard S, Djohan R et al. (2009) Near-total human face transplantation for a severely disfigured patient in the USA. Lancet 374: 203-209. doi:10.1016/S0140-6736(09)61155-7. PubMed: 19608265. 19608265 10.1016/S0140-6736(09)61155-7

[25] Strome M, Stein J, Esclamado R, Hicks D, Lorenz RR et al. (2001) Laryngeal transplantation and 40-month follow-up. N Engl J Med 344: 1676-1679. doi:10.1056/NEJM200105313442204. PubMed: 11386266. 11386266 10.1056/NEJM200105313442204

[26] Rispoli C, Andreuccetti J, Iannone L, Armellino M, Rispoli G et al. (2012) Anorectal avulsion: Management of a rare rectal trauma Int J Surg Case Rep 3. pp. 319-321. 10.1016/j.ijscr.2012.04.001 PMC3356549 22554940

[27] Eid KR, Costa G, Bond GJ, Cruz RJ, Rubin E et al. (2010) An innovative sphincter preserving pull-through technique with en bloc colon and small bowel transplantation. Am J Transplant 10: 1940-1946. doi:10.1111/j.1600-6143.2010.03167.x. PubMed: 20636461. 20636461 10.1111/j.1600-6143.2010.03167.x

[28] George AT, Dudding TC, Gurmany S, Kamm MA, Nicholls RJ et al. (2013) Pudendal Nerve Stimulation for Bowel Dysfunction in Complete Cauda Equina Syndrome. Ann Surg, Apr 10.[Epub ahead of print]. PubMed : 23579581 10.1097/SLA.0b013e31828e7602 23579581

[29] Ozkan O, Akar ME, Ozkan O, Erdogan O, Hadimioglu N et al. (2013) Preliminary results of the first human uterus transplantation from a multiorgan donor. Fertil Steril 99: 470-476. doi:10.1016/j.fertnstert.2012.09.035. PubMed: 23084266. 23084266 10.1016/j.fertnstert.2012.09.035

[30] Badylak SF, Weiss DJ, Caplan A, Macchiarini P (2012) Engineered whole organs and complex tissues. Lancet 379: 943-952. doi:10.1016/S0140-6736(12)60073-7. PubMed: 22405797. 22405797 10.1016/S0140-6736(12)60073-7

